# Understanding changes in dyspnoea perception in obstructive lung disease after mindfulness training

**DOI:** 10.1136/bmjresp-2018-000309

**Published:** 2018-06-23

**Authors:** Alice Malpass, Gene Feder, James W Dodd

**Affiliations:** 1 Centre for Academic Primary Care (CAPC), Bristol Medical School, University of Bristol, Bristol, UK; 2 Academic Respiratory Unit, University of Bristol, Southmead Hospital, Bristol, UK; 3 Academic Respiratory Medicine, University of Bristol, Bristol, UK

**Keywords:** perception of asthma/breathlessness, pulmonary rehabilitation, respiratory measurement

## Abstract

**Introduction:**

Dyspnoea has been defined as a ‘subjective experience of breathing discomfort that consists of qualitatively distinct sensations that vary in intensity’. However, the majority of available dyspnoea measures treat it as a single entity and rely on quantitative methodology. We propose that qualitative research can enhance our understanding of dyspnoea, in particular, how perception varies so much among patients with similar disease states. In this paper, we focus on how a specific type of inner attention—mindfulness—may alter perceptions of dyspnoea. The aim is to characterise mindfulness attention, which impacts on perceptions of dyspnoea and relate these to the multidimensional model of dyspnoea. We explore how an individual can change their perception and therefore relationship to similar disease states.

**Method:**

22 patients with asthma or chronic obstructive pulmonary disease were recruited from primary and secondary care to an 8-week course in mindfulness-based cognitive therapy (MBCT). 12 patients took part in an in-depth qualitative interview 2 months after completing the MBCT course. Data were recorded, transcribed and then analysed using a framework approach, drawing on components of the multidimensional model of dyspnoea (multidimensional dyspnoea profile, MDP).

**Results:**

We found that MBCT training involves developing three types of mindful attention (broad attention, informative attention and re-directive attention), which impact on perceptions of the sensory dimension of dyspnoea. MBCT appears to target affective and sensory perceptions articulated in the MDP model.

**Conclusion:**

More research is needed into how mindfulness-based interventions may mediate the relationship between affective experience and the sensory perception of dyspnoea symptoms.

**Key messages**

- Why does perception of dyspnoea vary so much among respiratory patients with the same disease state?

- We know that the perception of dyspnoea is a complex individual interpretative process of sensory input highly influenced by many non-sensory factors such as attention.

- The findings show mindfulness training involves developing different types of non-evaluative attention of interoceptive (internal) sensations, which over time may mediate perceptions of these sensations.

## Introduction

The experience of dyspnoea among patients living with a respiratory disease can lead to increased stress, distress, anxiety and depression.^1–3^ Emotional states shape the quality and intensity of dyspnoea, though the exact cause relationship and direction of causality between dyspnoea and anxiety are unclear.^3 4^ Studies from the general population investigating the role of anxiety in reporting dyspnoea are similar to findings among respiratory disease populations: stratifying by lung function, dyspnoea is more common among people with anxiety symptoms.^5^ However, despite suggestions of a causal relationship, such as the dyspnoea–anxiety–dyspnoea cycle,^6^ the literature remains inconclusive with Leviseth and colleagues arguing that “the cause and effect relationship between anxiety and dyspnoea may differ depending on the situation in which dyspnoea is experienced” (p. 1155).^5^

Mindfulness training may benefit respiratory patients who experience anxiety and depression.^7^ It is a non-pharmacological approach^8^ that promotes self-management.^9^ A recent trial found significant improvement in emotional function among patients with chronic obstructive pulmonary disease (COPD)^10^ and a trial with patients with asthma found clinically significant improvements in asthma-related quality of life and stress.^11^ While individual studies have shown psychological benefit, a recent systematic review of the effectiveness of mindfulness in people with a respiratory diagnosis was inconclusive, largely due to inconsistencies in study methodologies.^1^

Qualitative research has shown that an 8-week course in mindfulness led to patients being able to notice subtle bodily sensations, without a negative cognitive–evaluative response, and detection of early warning signs of dyspnoea, critical to effective self-management.^12^ One barrier in measuring the impacts of psychological interventions such as mindfulness on the experience of dyspnoea is the challenge of characterising dyspnoea.

### Different dyspnoeas

The American Thoracic Society currently defines dyspnoea as a ‘subjective experience of breathing discomfort that consists of qualitatively distinct sensations that vary in intensity’^13^; put simply, ‘there are different dyspnoeas’.^14^ A more systematic articulation of the ‘language of dyspnoea’ has only developed within the last 15 years.^15^ Part of the variation in the experience of dyspnoea, how it is expressed and described by patients, is related to the complexity of its perception: “…(T)he perception of dyspnoea is a complex individual interpretation process of sensory input that is highly influenced by many nonsensory factors such as attention, emotions, motivation, memory, personality, expectation or prior experience” (p. 840).^16^ As with the perception of pain, which is modulated by attention (p. 841),^16^ the perception of dyspnoea may also be sensitive to attention. In this paper, we focus on a specific type of inner attention—mindfulness—hypothesising how a training of attention can contribute to re-perceiving the experience of breathing and breathlessness.

### Sensory and somatic aspects of experience

**Figure 1:**
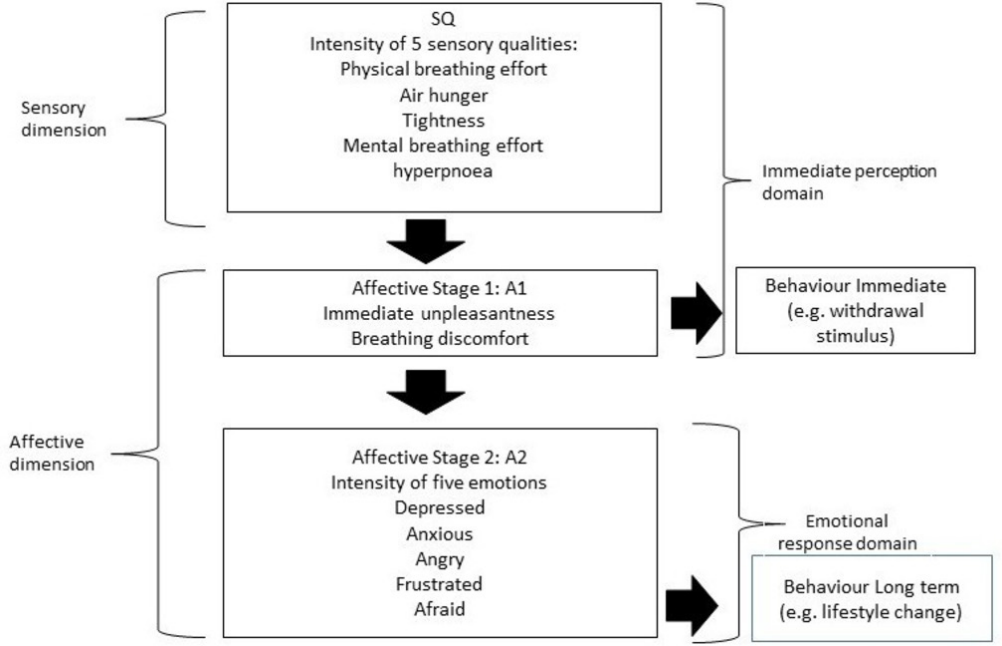
The multidimensional dyspnoea profile model (Banzett *et al*^17^).

Until recently, most measurements of dyspnoea treated it as a single entity. Dyspnoea is the result of a complex interaction of physiological, psychosocial, social and environmental factors. The recent multidimensional model for dyspnoea (multidimensional dyspnoea profile, MDP)^17^ suggests that dyspnoea, like pain, comprises multiple components that can be measured as different entities. Functional brain imaging studies of dyspnoea suggest a common neurophysiology of dyspnoea and pain.^17^ Like pain, dyspnoea has at least two distinct separate dimensions: sensory and affective. The sensory dimension includes sensory intensity and sensory quality (as well as location and time course of sensation). The affective dimension splits into immediate unpleasantness (stage 1) and emotional response and evaluation (stage 2) (see figure 1). Three distinct sensations of dyspnoea have been described: air hunger, work/effort and chest tightness. The multidimensional model of dyspnoea is intended to help understand the actions of therapeutic interventions such as mindfulness approaches.^14^

### Mindfulness training and the breath

In this paper, we report further analysis of qualitative data collected during a feasibility study of mindfulness -based cognitive therapy (MBCT) for patients living with COPD or asthma.^12^ MBCT, a psychological approach to coping with difficulty, is a fusion of meditation and mindfulness practices (derived from the insight meditation tradition) with cognitive–behavioural therapy methods.^18^ MBCT is a manualised programme, delivered as 8-weekly 2-hour sessions.^19^ In each session, participants explore a different theme of mindfulness. For example, in week 1, participants explore the role that ‘automatic pilot’ plays on their mood, thoughts and daily activities. The term ‘automatic pilot’ describes a ‘state of mind in which one acts without conscious awareness of sensory perception’ (p. 21).^18^ The MBCT method involves focusing attention on the breath as the first step in noticing and observing habitual thoughts, emotions and behaviours. Participants are taught how to unravel their ‘bundle’ of difficult experience (such as an episode of dyspnoea or anxiety). Participants identify their unique mood ‘signature’, that is, the early warning signs when they are beginning to become anxious or feel distressed about their breathing. The course intention is for participants to realise they have choices in how they can respond to difficulty instead of what may be their learnt maladaptive habitual reactions. As the weeks progress, participants learn to relate differently to difficult sensations, feelings and thoughts.

In this paper, we use a conceptual model to explain the marked differences *within* the individual patient’s experience and response to dyspnoea after an 8-week training in mindfulness and how a patient comes to experience a ‘different kind of breath’ when there has been no objective change in the physiology underlying their dyspnoea. Our aim in this paper is to characterise three types of mindfulness attention that impact on perceptions of dyspnoea and relate these to the multidimensional model of dyspnoea.^14^ By exploring how an individual can change their perception and therefore relationship to the same disease symptoms, we hope to improve our understanding of how perception of dyspnoea varies so much among patients with similar disease states.

In our discussion, we link three types of mindful attention identified in our data with the growing body of work within cognitive psychology on the links between mindfulness training, modes of attention and well-being.^20 21^ We end our discussion with reference to the interoception literature, in particular the links between interoceptive awareness (of respiratory sensations) with attentional training.^22^

## Methods

### Data collection

A total of 22 patients were recruited from primary and secondary care for an 8-week course of MBCT, delivered in a community setting. They were clinically stable and free from exacerbations during the course of the study. Of the 22 patients who attended the course, 12 patients were purposively sampled to take part in an in-depth qualitative interview 2 months after completion. We also collected enquiry data from each weekly session. Enquiry data refers to the discussions that took place between participants and the mindfulness teachers after each of the mindfulness practices led each week. Verbatim enquiry and interview data were recorded, transcribed and analysed.

### Analysis

The previous analysis of the interview data was thematic, characterising the overall patient experience of mindfulness training and its impact on living with COPD or asthma.^12^ In this paper, our analysis of interview data is based on a conceptual framework: the multidimensional model of dyspnoea. We have also included analysis of enquiry data (not analysed previously). Within a framework analysis,^23^ we used a multidimensional model (see figure 1) to organise the verbatim data in the appropriate column. This included descriptions of sensory intensity, immediate unpleasantness, emotional responses, immediate (reactive) behaviours and long-term behavioural changes (lifestyle changes). We then used the framework method to explore the concordance between different types of mindfulness attention and the MDP model. This allowed us to build up examples of different types of mindfulness attention and see their relationship to the components of the MDP model. We also scrutinised the data set for disconfirming cases.

## Findings

We found three types of mindfulness attention, which impacted on perceptions of dyspnoea: (1) *broad attention* that is being cultivated through mindfulness practices; (2) *informative bare attention* related to an increase in perceptual awareness as opposed to habitual conceptual/cognitive awareness; (3) *flexibility in attention*, so participants can re-direct their attention to their breath beyond the anatomic apparatus of breathing. In discussing each attentional training type, we explore how it relates to the multidimensional model of dyspnoea.

### Broad attention: non-identification with sensory experience

Training in developing a broad attention is particularly important for participants being able to feel separate or distance themselves from the unpleasant experience of dyspnoea, depicted in the MDP model as Affective Stage 1:

My chest was getting tighter in a kind of asthma way but I was able to distance myself from a sense of panic. Where I would normally have gone, need to go for my inhaler so quickly, I was quite aware of it, being able to be removed from it at the same time. It was almost like I was separate to it in a way… the tightness was across the top of my chest, both left and right and it feels as though everything is closing a little bit in, almost like my lungs are getting smaller. I had awareness of my body and then awareness of being sort of separate to it, more like a sense of space around it. I wasn’t panicking, just feel it, notice it, let it be and just accept it almost as a sensation, don’t try to fight it or panic and by just accepting it I then sort of felt (I was) moving away from the sensation a little bit, not focusing on it too much, just enough awareness. (Asthma, F, Inquiry data, wk 2)

This participant with asthma describes an attention that is distant and aloof. She is able to find distance from the experience while describing the detail of the sensations. Speeth talks about ‘witnessing, as if from above’ (p. 98) to convey this sense of breadth in attention.^24^

The breadth of awareness allows an attention that has a light touch and does not stick (with automatic negative thoughts and emotions) to what it is noticing:

Even though there are parts of me that I know are in pain (in my chest), *I don’t stick to that any more*. Whereas the first week it was like, oh that really really hurts, now I just go there, accept it and move on. I’m not dwelling on it. Whereas before I was always in it, I don’t even look for it now. *I give it a little bit of attention and then I just move on*. (Asthma, M, Inquiry data, wk 3).

Another way to understand broad attention is in relation to what Speeth describes in psychotherapy as ‘panoramic attention’ (as opposed to one-pointed attention): ‘a feeling of impartiality, of spaciousness, of breadth of vision’ (p. 151).^24^ By broadening our attention, dyspnoea is no longer an encompassing experience, which eliminates awareness of everything else and becomes the way patients identify themselves in that moment. Instead, the breadth of attention reduces identification. Developing a de-centred approach makes it possible to take the dyspnoea less personally, to relate to dyspnoea as an event that arises and will pass away.

This aspect of attention relates to various components of the multidimensional model. Dyspnoea is not a single sensation; at least three distinct sensations have been proposed including air hunger, work/effort and chest tightness. The above accounts describe sensations of chest tightness. The multidimensional model of dyspnoea has two distinct dimensions: sensory and affective. The affective components split into an immediate unpleasantness (stage 1) and emotional response and evaluation (stage 2). In the above examples, we can see how participants are aware of the sensory aspects of their experience but are able, through a breadth of attention, to influence their emotional response and evaluation of that experience.

### Informative bare attention: being with the detail of sensory experience

Learning the skill of ‘being with’ the immediate sensory experience without adding a proliferation of thoughts about how frightening or anxiety provoking the sensation feels is possible for patients with COPD:

There’s times when you think ‘I’m not going to breathe again’. And that makes it worse. If you can sit and say, ‘oh that’s a bit breathless, oh, here we are then’, you’re fine. But if you think ‘oh my god, I’m not going to breathe again!’ it doesn’t help you one little bit. You just get worse. (COPD, F, Interview data)

This participant’s ability to be present with her experience of dyspnoea is based on her mindfulness training, in surrendering to experience without trying to change, fight or fix it, characterised by her phrase: ‘here we are then’. The participant has gained understanding of how thoughts and feelings are reactions to sensations and how catastrophising thoughts feed a sense of dyspnoea: ‘it doesn’t help, you just get worse’. In mindfulness, this quality of awareness is often called ‘bare attention’, an attention that is ‘bare of labels’.^24^ Labels refer to the thoughts that attach themselves to experience, such as ‘I’m not going to breathe again’ and instead experiences dyspnoea as dyspnoea: ‘oh, that’s a bit breathless, here we are then’.

The bare attention involves noticing the detail of sensation without it becoming an attention that is hypervigilant or an anxious watching:

When we get asthma there’s a lot of mucus production going on in the throat. I’ve always imagined that that was in my chest. And that my little bronchia’s were being filled up with loads of mucus. But I’ve noticed during this meditation that by choosing to breathe through my nose rather than my mouth, I bypass the problems that that usually triggers. I was able to just be with the feeling of mucus on my throat without it triggering any kind of asthma attack like it normally would. The sensation is here (touching throat) and not in there (touching chest) as I imagined it was always happening. (Asthma, F, Inquiry data, wk 7)

Through mindful awareness, the participant has gained new information about the sensory aspect of her experience, allowing her to reconfigure her experience of the breathing discomfort, rather than how she had imagined the sensory dimension and breathing discomfort to be: ‘I’d always imagined that was in my chest’.

Similarly, one participant described being able to identify an earlier warning sign in the body that a breathless episode was threatening to develop:

The hospital has always said you will start to feel yourself going tight. I found, before I go tight, my shoulders go up which causes the tightness to your chest. You start to panic. I haven’t done that for ages. I’ve realised, pull your shoulders down, it bloody stops it (tightness in chest)! (COPD, M, Interview data)

Participants with COPD and asthma learnt to identify subtle body sensations that previously they had not been aware of. In the MDP model, the sensory dimension and breathing discomfort are aligned as the immediate perception domain. In the examples explored above, mindfulness training in a type of informative attention begins with detailed noticing of the immediate sensory experience. In terms of the MDP model, attending mindfully to sensory experience facilitates changes in both the immediate perception domain, with reductions in the immediate unpleasantness of sensations, as well as influencing changes in the emotional response domain—such as reductions in reported anxiety and panic.

### Re-directing attention to alternate sensory experience

In MBCT, participants are invited to experience their breath beyond the normal respiratory anatomical structures, to feel the movements of the breath in their lower belly and back. For people with chronic respiratory symptoms, this was initially challenging, but became a way to re-direct attention away from feelings of constriction in their chest:

I was surprised about the amount of breathing I could actually feel coming from my tummy. At the start I was a bit nervous about trying to focus on that area. But the more I tried to feel what was coming from my tummy, I could actually feel something. It was almost blocking off of anything (unpleasant) I could feel coming from the chest. I go (to my belly) automatically now. I just do it without thinking. It’s a different kind of breath. (Asthma, F, Inquiry data, wk 8)

Mindfulness training teaches participants to notice their sensory experience of breathing in ways they may not have explored before. This new information about the sensory experience of their breathing impacts on how they experience and relate to their dyspnoea:

I was on loads of inhalers in week 1. I don’t take my inhaler daily anymore. I just take it two or three times a week. I’ve not really had to grab it at all. When you first asked us to breathe into our bellies, I was a bit, ‘I can’t do that’. It was all an effort. I just feel my lungs are a lot clearer and I’m taking a lot of pressure off this top bit (points to chest). I feel that my breath goes all the way through (to the belly), rather than just here (stopping at the diaphragm). (Asthma, F, Inquiry data, wk 7)

For some participants, being asked to imagine the breath going beyond the pharynx, trachea and lungs and notice movements of the breath in the lower abdomen facilitates a new relationship with the breath and dyspnoea.

For other participants, focusing on their breathing in their abdomen intensified the experience of discomfort in their chest. These participants still used the mindfulness skill of re-directing attention, but instead of using sensations of abdominal breathing, they chose another part of the body:

The bit I found most difficult was concentrating on my breathing. As I did, it became more difficult, I felt as though I was hungry for air. I kept thinking, your breath is failing, it’s uncomfortable. But it was only uncomfortable when I was thinking about it. I could feel my tummy moving up and down but the tightness was here (across chest). An incomplete feeling, I wanted to gasp. I thought it the most bizarre place (to concentrate on my knees) but it just worked, it (the distress) went immediately. I just pictured both of my knees, the cartilage and the muscle, that stretch and immediately the tension went from my chest. I stopped thinking about it. (COPD, F, Inquiry data, wk 3)

Focusing attention on bodily sensations in the knees leaves less space for rumination and catastrophising thoughts about dyspnoea. It redirects attentional resources away from ruminative thinking.^25^ In terms of the MDP model, focusing on another sensory dimension (in the knees) rather than the sensations of air hunger and chest tightness had a calming effect on the breath: “I pictured both my knees… immediately the tension went from my chest”.

Similarly, for another participant, re-directing their attention to noticing sensations in other parts of the body helped relieve perceptions of tightness in the chest:

(T)hat was the connection that I made right at the beginning. Doing that body scan enabled me to take the emphasis from my chest onto somewhere else. And I find that my chest then functions good on its own. (COPD, M, Interview data)

## Discussion

In the MDP model, it is assumed that as behavioural approaches largely focus on patients’ cognition and emotion, they will predominantly alter Affective Stage 2 (emotional response to dyspnoea) and sometimes Affective Stage 1 (immediate unpleasantness)^16^ (see figure 1). We have found that mindfulness training can also have an impact on perceptions of the sensory dimension of dyspnoea. This may be because mindfulness training is centred on the body, with a large proportion of the 8-week course focused on training attention on bodily sensations.

**Figure 2:**
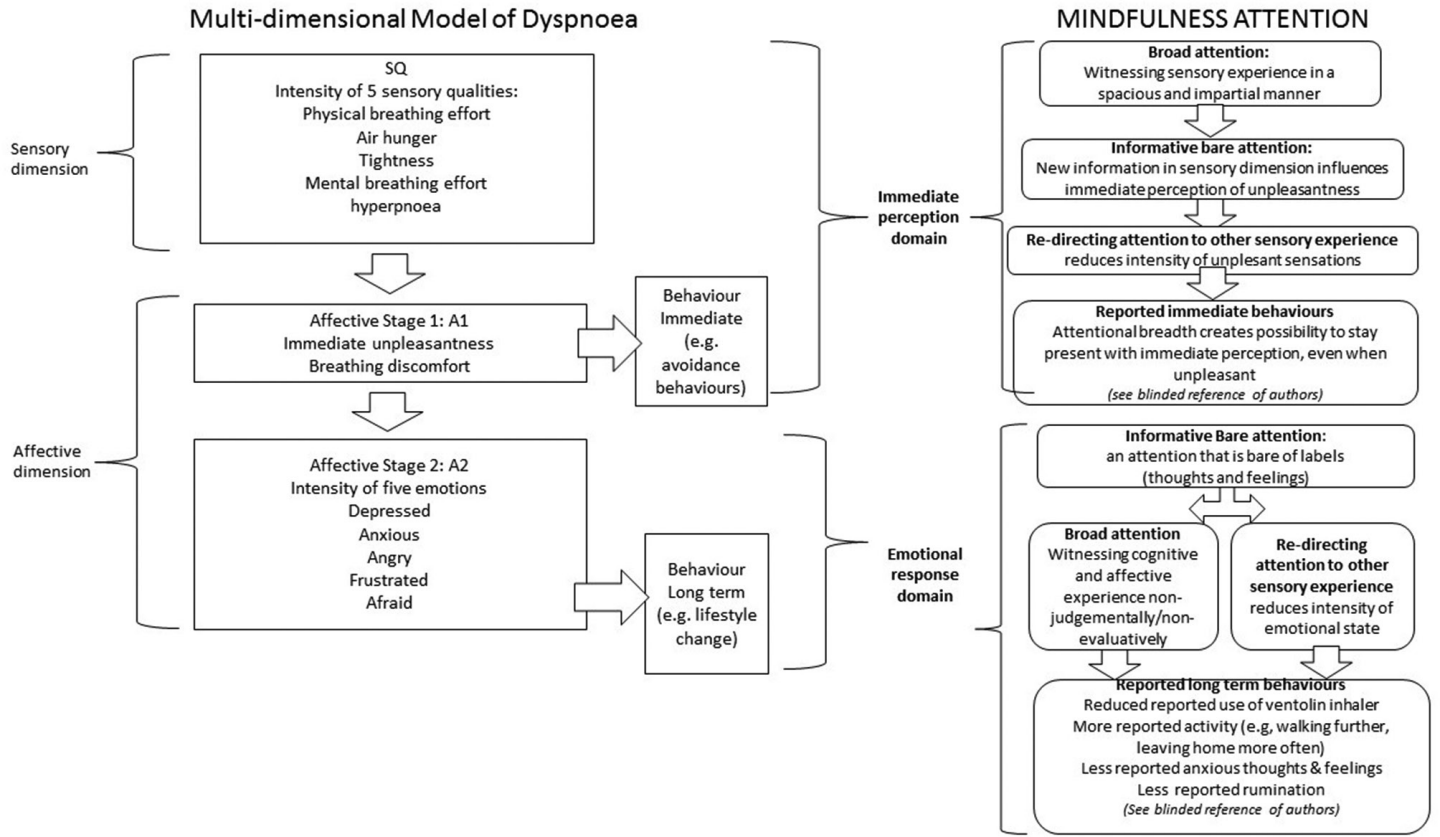
How attentional training in mindfulness align with the multidimensional model of dyspnoea.

Figure 2 shows how training in mindfulness attention aligns with the components of dyspnoea underlying the MDP model. In a previous analysis of our data, we concluded that mindfulness may help respiratory patients by removing the psychological barriers to being more active, that is, working on the emotional and cognitive response to dyspnoea. By re-analysing our data through the lens of the MDP model, we have refined our understanding of patient-reported benefits and moved beyond our original interpretations of the interviews with MBCT participants’ data. We know that the perception of dyspnoea is a complex individual interpretative process of sensory input highly influenced by many non-sensory factors such as attention (p. 840).^16 26^ In figure 2, we suggest how broad attention impacts on the immediate sensory perception, giving rise to new information about the sensory experience itself, which in turn impacts the immediate experience of unpleasantness. In the Affective Stage 2 of the MDP, all three types of mindful attention (broad attention, informative attention and redirecting attention) are influential in reducing the intensity of emotions experienced.

### How our findings relate to existing literature on dyspnoea

Training in ‘receptive attention’ is at the core of mindfulness, alongside cultivating a certain intention and attitude that underlie that attention.^27^ A large review concluded: ‘relatively few empirical evaluations of meditation and attention have been conducted’ (p. 200).^28^ Interest in interoceptive attention has grown substantially since the publication of this review^29^ including cognitive studies of attentional awareness in mindfulness training,^30^ as well as more theoretical work looking at attention as a central mechanisms of action in mindfulness training.^31 32^ Much of this work concludes that mindfulness training of attention skills underpins emotional and cognitive flexibility, bringing about the ability to maintain non-judging awareness of feelings, thoughts and experiences.^33^ This body of work aligns with our own interest in the role of attention in perceiving dyspnoea. We believe that our analysis moves forward our understanding by exploring how different types of mindfulness attention are described and experienced by respiratory patients as they come to re-perceive their dyspneoa at the immediate sensory level of the body.

The Liverpool and other models of mindfulness^34–36^ focus on effortful attentional skills. The three types of attention we identify—broad attention, informative bare attention and re-directional attention—would all be classed as examples of effortful attentional regulation, which come about through training in sustaining, monitoring, disengaging and shifting attentional processes. Shapiro *et al* maintain that mindful attention results in the enhancement of three types of attentional skills from cognitive psychology: vigilance or sustained attention, including the capacity to attend for long periods of time to one object; switching attention, including the ability to shift the focus of attention between objects or mental sets at will; and cognitive inhibition, the ability to inhibit secondary elaborative processing of thoughts, feelings and sensations.^37^

Consistent with the concept of attentional skill from cognitive psychology, Bailey also describes *controlling a perception* of dyspnoea, whereas we frame the training of attention in mindfulness leading to *a re-perceiving* of dyspnoea.^6^

Our findings are supported by other qualitative work, which also suggests mindfulness training has an impact on perceptions of dyspnoea.^7^ The underlying psychophysiological mechanisms of such a shift in perception of dyspnoea from training in different types of mindful attention are likely to be complex. If we take one of the mindful types of attention—re-directional attention—we can begin to link our findings to existing research, which distinguishes between the intensity and unpleasantness of dyspnoea.

In a discussion of the affective components of dyspnoea, researchers argue that directing a patient’s attention *away* from respiratory sensation decreases unpleasantness but not intensity.^3^ Indeed, studies using attentional distraction to reduce perceived exertional dyspnoea in COPD have been inconclusive^38^ and are not recommended in American Thoracic Society Guidelines.^13^ However, in our study some participants re-directed their attention *away* from the place where the dyspnoea felt most unpleasant or intense (such as the chest) and *towards* another part of the body where they could feel the sensations of their breath (such as the belly). We also have examples of participants taking their attention away from sensations of the breath altogether (such as to the knees), though this was more uncommon. The re-directing of attention away from sensations of the breath in certain parts of the body within MBCT is distinct from distraction techniques.^39^ Within MBCT, re-directing attention is the result of an accepting mode of response, in which patients are trained to become less aversive towards the sensations of dyspnoea and any distressing thoughts about dyspnoea, to notice their occurrence without attempting to alter their content or frequency. There is also clinical merit in MBCT, enabling patients with asthma to consider the anatomical focus of their dyspnoea, for example, to the large airway and away from the chest. This may reflect the presence of vocal cord dysfunction, a term that refers to inappropriate adduction of the vocal cords while breathing. This functional disorder is as an important mimic of asthma and can lead to inappropriate treatment with potentially harmful drugs including high-dose steroids. A study^40^ with 31 patients with COPD showed that a psychological intervention focused on helping patients with COPD reduce anxiety and manage their dyspnoea *did* reduce the intensity of dyspnoea during resistive loading 6 months after the intervention. Of particular interest is that the intervention was cognitive–behavioural therapy (CBT) based, which has similarities as well as clear differences to MBCT. For example, in the CBT intervention, patients kept a daily breathing record with the aim of increasing early awareness of dyspnoea and recognising unhelpful cognitions that increased anxiety and prompting behavioural management strategies before dyspnoea intensified. The behavioural aspects included ‘replacing cognitions with more realistic, coping orientated ones’ and ‘pursed lip breathing’ (p. 41).^40^ While patients in our study identified early warning signs of dyspnoea and unhelpful cognitions, rather than challenging or replacing cognitions, MBCT trains patients in an accepting mode of response,^18^ encouraging an attitude of letting go and reductions in identification with cognitions. While both CBT and MBCT encourage noticing and monitoring dyspnoea, they differ in the attitude with which they notice and monitor: CBT favours replacing cognitions, whereas MBCT favours an attitude of non-identification with cognitions. This difference in attitude gives rise to different ways of managing unhelpful dyspnoea-related cognitions. Nevertheless, both MBCT and CBT may help increase realistic symptom appraisal, thereby reducing dyspnoea-related anxiety.

For people living with COPD and anxiety, research has shown that meditation may improve their ability to detect and monitor respiratory load and immediate ventilatory needs.^10^ This may be because mindfulness training cultivates a non-evaluative awareness of interoceptive (internal) sensations, which over time may mediate perceptions of these sensations.^41^ Pilot work suggests that greater respiratory interoceptive accuracy (ie, perceiving internal sensations of dyspnoea accurately) was related to less anxiety among participants practising mindfulness meditation compared with control participants.^41^ Instead of using the term *non-evaluative*, we have described a bare attention, an attention without labels. Daubenmier and colleagues make an explicit connection between the type and quality of attention and the potential for respiratory interoceptive accuracy.^41^ Our main finding is this connection between non-evaluative attentional training and sensory perception.

Mehling and colleagues remind us that interoceptive awareness has not always been regarded as a good thing. It is only in recent years that the ability to recognise subtle bodily cues has been shown to be beneficial for managing chronic illness and mental health conditions.^31^ Our finding of three types of attention in the re-perceiving of dyspnoea-related bodily sensations concurs with more theoretical work that shows the degree to which interoceptive awareness is beneficial or maladaptive depends on ‘distinct modes of mind’.^42^ For example, we have shown examples in our findings of how bare informative attention involves noticing the detail of sensation without it becoming an attention that is hypervigilant because it is ‘bare of labels’. This description concurs with the distinction made in the literature between two modes of mind-focusing attention on immediately experienced sensations (which is adaptive) versus an abstract ruminative self-focus (which is maladaptive). Our focus on three types of attention will be of interest to those researching how different types of attention help distinguish beneficial and maladaptive forms of body awareness.

### Limitations and further research

A limitation of this study is that we have not measured the components of MDP in our participants before and after the mindfulness training, which would be a test of the effect of the intervention on dyspnoea using the multicomponent model. However, the intervention took place in 2010–2011 predating by 4 years publication of the MDP. Further research is now needed that uses the MDP as an outcome measure for mindfulness interventions so the relationship between different types of attention and the MDP can be explored further.

Our methodological approach is unique in focusing on first-hand accounts of changes in attention and interoceptive awareness of sensations of dyspnoea in order to understand reported changes in perception of dyspnoea. Recent research distinguishes between interoceptive accuracy (which is assessed via objective tests) and interoceptive sensibility (which probes for perceived aptitude to notice bodily sensations).^43^ Both are important to measure separately. A strength of our qualitative approach then is that it explores in depth aspects of ‘interoceptive sensibility’, described as ‘the self-perceived dispositional tendency to be internally self-focused’ (p. 67).^43^ Further research should use measurements of interoceptive accuracy and interoceptive sensibility together as mediators of an MBCT intervention for respiratory patients.^30^

The influence of emotional states on symptoms perception in respiratory disease is a topic of growing interest to researchers and clinicians.^4^ Further research is needed to explore how mindfulness-based interventions may mediate between affective experience and the sensory perception of dyspnoea symptoms. A recent study exploring the tendency of patients with COPD to experience an exacerbation shows dyspnoea perception was greater in frequent exacerbators compared with infrequent and that infrequent exacerbators had a blunted response compared with controls.^44^ Ridsdale and Hurst^45^ suggest if patients with altered perceptions of dyspnoea can be identified, then ‘interventions (such as) learnt breathing techniques just might help and may be more likely to help than say, inhaled corticosteroids’ (p. 107).

MBCT as an intervention, while not teaching specific breathing techniques, does teach three types of attention, which seem to influence perceptions of the breath and dyspnoea. Future research should test the hypothesis that (1) MBCT could help infrequent exacerbators develop ‘less blunted responses’ to dyspnoea perception by becoming more aware of internal sensations of dyspnoea and (2) that MBCT could help frequent exacerbators re-appraise sensory perception of dyspnoea symptoms, by developing a mindful attention that is not hypervigilant and is decoupled from unhelpful emotions and cognitions.

## Conclusions

MBCT appears to target affective and sensory perceptions outlined in the MDP. The findings reported here increase our understanding of differences in dyspnoea for the similar disease states. More research is needed into how mindfulness-based interventions may mediate between affective experience and the sensory perception of dyspnoea symptoms. Now that we have a more robust measurement of dyspnoea with the MDP, future research could include a clinical trial of MBCT in COPD and asthma with perceptions of dyspnoea as the primary outcome and measurements of interoceptive awareness (using the MAIA) as a mediating factor.

## References

[1] Harrison SL, Lee A, Janaudis-Ferreira T, et al Mindfulness in people with a respiratory diagnosis: a systematic review. Patient Educ Couns 2016;99:348–55. 10.1016/j.pec.2015.10.013 26561308

[2] Chetty U, McLean G, Morrison D, et al Chronic obstructive pulmonary disease and comorbidities: a large cross-sectional study in primary care. Br J Gen Pract 2017;67:e321–8. 10.3399/bjgp17X690605 28450344 PMC5409435

[3] Scano G, Gigliotti F, Stendardi L, et al Dyspnea and emotional states in health and disease. Respir Med 2013;107:649–55. 10.1016/j.rmed.2012.12.018 23347530

[4] von Leupoldt A, Chan PY, Bradley MM, et al The impact of anxiety on the neural processing of respiratory sensations. Neuroimage 2011;55:247–52. 10.1016/j.neuroimage.2010.11.050 21111831 PMC3031667

[5] Leivseth L, Nilsen TI, Mai XM, et al Lung function and anxiety in association with dyspnoea: the HUNT study. Respir Med 2012;106:1148–57. 10.1016/j.rmed.2012.03.017 22579439

[6] Bailey PH The dyspnoea-anxiety-dyspnoea cycle—COPD patients’ stories of dyspnoea:“It’s scary/when you can’t breathe”. Qualitative health research 2004;14:760–78. 15200799 10.1177/1049732304265973

[7] Benzo R Mindfulness based stress reduction in severe COPD: pilot on feasibiity, effect on quality of life and qualitative analysis. Am J Respir Crit Care Med 2011;183:A1456.

[8] Jones D Chronic obstructive pulmonary disease part 2: non-pharmacological therapy. Nurs Stand 2015;29:53–8. 10.7748/ns.29.34.53.e9671 25902253

[9] Benzo RP Mindfulness and motivational interviewing: two candidate methods for promoting self-management. Chron Respir Dis 2013;10:175–82. 10.1177/1479972313497372 23897933 PMC3953462

[10] Chan RR, Giardino N, Larson JL A pilot study: mindfulness meditation intervention in COPD. Int J Chron Obstruct Pulmon Dis 2015;10:445 10.2147/COPD.S73864 25767382 PMC4354397

[11] Pbert L, Madison JM, Druker S, et al Effect of mindfulness training on asthma quality of life and lung function: a randomised controlled trial. Thorax 2012;67:769–76. 10.1136/thoraxjnl-2011-200253 22544892 PMC4181405

[12] Malpass A, Kessler D, Sharp D, et al MBCT for patients with respiratory conditions who experience anxiety and depression: a qualitative study. Mindfulness 2015;6:1181–91. 10.1007/s12671-014-0370-7

[13] Parshall MB, Schwartzstein RM, Adams L, et al An official American Thoracic Society statement: update on the mechanisms, assessment, and management of dyspnea. Am J Respir Crit Care Med 2012;185:435–52. 10.1164/rccm.201111-2042ST 22336677 PMC5448624

[14] Lansing RW, Gracely RH, Banzett RB The multiple dimensions of dyspnea: review and hypotheses. Respir Physiol Neurobiol 2009;167:53–60. 10.1016/j.resp.2008.07.012 18706531 PMC2763422

[15] Williams M, Cafarella P, Olds T, et al The language of breathlessness differentiates between patients with COPD and age-matched adults. Chest 2008;134:489–96. 10.1378/chest.07-2916 18490404

[16] von Leupoldt A, Seemann N, Gugleva T, et al Attentional distraction reduces the affective but not the sensory dimension of perceived dyspnea. Respir Med 2007;101:839–44. 10.1016/j.rmed.2006.06.033 16971103

[17] Banzett RB, O’Donnell CR, Guilfoyle TE, et al Multidimensional Dyspnea Profile: an instrument for clinical and laboratory research. Eur Respir J 2015;45:1681–91. 10.1183/09031936.00038914 25792641 PMC4450151

[18] Crane R Mindfulness-based cognitive therapy: distinctive features. Routledge 2013.

[19] Segal Z, Williams M, Teasdale J Mindfulness based cognitive therapy: a new approach to preventing relapse. 2nd edition: Guildford Press, 2012.

[20] Jensen CG, Vangkilde S, Frokjaer V, et al Mindfulness training affects attention—or is it attentional effort? J Exp Psychol Gen 2012;141:106 10.1037/a0024931 21910559

[21] Jha AP, Krompinger J, Baime MJ Mindfulness training modifies subsystems of attention. *Cognitive, Affective, &*. Behavioral Neuroscience 2007;7:109–19. 10.3758/cabn.7.2.109 17672382

[22] Farb N, Mehling WE Interoception, contemplative practice, and health. Front Psychology 2016;7:1898. 10.3389/fpsyg.2016.01898 PMC5131001 27990133

[23] Ritchie J, Spencer L Qualitative data analysis for applied policy research. The qualitative researcher’s companion 2002:305–29.

[24] K.R S On Psychotherapeutic attention. Journal of Transpersonal Psychotherapy 1982;14/2:141–59.

[25] Williams JM Mindfulness and psychological process. Emotion 2010;10:10(1), 1 10.1037/a0018360 20141295

[26] Paulus MP The breathing conundrum-interoceptive sensitivity and anxiety. Depress Anxiety 2013;30:315–20. 10.1002/da.22076 23468141 PMC3805119

[27] Brown KW, Ryan RM, Creswell JD Mindfulness: Theoretical foundations and evidence for its salutary effects. Psychological inquiry 2007;18:211–37.

[28] Cahn BR, Polich J Meditation states and traits: EEG, ERP, and neuroimaging studies. Psychol Bull 2006;132:180 10.1037/0033-2909.132.2.180 16536641

[29] Farb NA, Anderson AK, Segal ZV The mindful brain and emotion regulation in mood disorders. Can J Psychiatry 2012;57:70–7. 10.1177/070674371205700203 22340146 PMC3303604

[30] de Jong M, Lazar SW, Hug K, et al Effects of mindfulness-based cognitive therapy on body awareness in patients with chronic pain and comorbid depression. Front Psychol 2016;7 10.3389/fpsyg.2016.00967 PMC4927571 27445929

[31] Mehling WE, Price C, Daubenmier JJ, et al The Multidimensional Assessment of Interoceptive Awareness (MAIA). PLoS One 2012;7:e48230 10.1371/journal.pone.0048230 23133619 PMC3486814

[32] Lutz A, Slagter HA, Dunne JD, et al Attention regulation and monitoring in meditation. Trends Cogn Sci 2008;12:163–9. 10.1016/j.tics.2008.01.005 18329323 PMC2693206

[33] Chiesa A, Malinowski P Mindfulness-based approaches: are they all the same? J Clin Psychol 2011;67:404–24. 10.1002/jclp.20776 21254062

[34] Malinowski P Neural mechanisms of attentional control in mindfulness meditation. Front Neurosci 2013;7:8 10.3389/fnins.2013.00008 23382709 PMC3563089

[35] Hölzel BK, Lazar SW, Gard T, et al How does mindfulness meditation work? Proposing mechanisms of action from a conceptual and neural perspective. Perspect Psychol Sci 2011;6:537–59. 10.1177/1745691611419671 26168376

[36] Slagter HA, Davidson RJ, Lutz A Mental training as a tool in the neuroscientific study of brain and cognitive plasticity. Front Hum Neurosci 2011;5:5 10.3389/fnhum.2011.00017 21347275 PMC3039118

[37] Shapiro SL, Carlson LE, Astin JA, et al Mechanisms of mindfulness. J Clin Psychol 2006;62:373–86. 10.1002/jclp.20237 16385481

[38] Livermore N, Sharpe L, McKenzie D Selective attention to threatening information in anxious patients with chronic obstructive pulmonary disease. Cognitive Therapy and Research 2007;31:885–95.

[39] Najmi S, Riemann BC, Wegner DM Managing unwanted intrusive thoughts in obsessive-compulsive disorder: relative effectiveness of suppression, focused distraction, and acceptance. Behav Res Ther 2009;47:494–503. 10.1016/j.brat.2009.02.015 19327753

[40] Livermore N, Dimitri A, Sharpe L, et al Cognitive behaviour therapy reduces dyspnoea ratings in patients with chronic obstructive pulmonary disease (COPD). Respir Physiol Neurobiol 2015;216:35–42. 10.1016/j.resp.2015.05.013 26049126

[41] Daubenmier J, Sze J, Kerr CE, et al Follow your breath: respiratory interoceptive accuracy in experienced meditators. Psychophysiology 2013;50:777–89. 10.1111/psyp.12057 23692525 PMC3951998

[42] Watkins E, Teasdale JD Adaptive and maladaptive self-focus in depression. J Affect Disord 2004;82:1–8. 10.1016/j.jad.2003.10.006 15465571

[43] Garfinkel SN, Seth AK, Barrett AB, et al Knowing your own heart: distinguishing interoceptive accuracy from interoceptive awareness. Biol Psychol 2015;104:65–74. 10.1016/j.biopsycho.2014.11.004 25451381

[44] Scioscia G, Blanco I, Arismendi E, et al Different dyspnoea perception in COPD patients with frequent and infrequent exacerbations. Thorax 2017;72:117–21. 10.1136/thoraxjnl-2016-208332 27586869

[45] Ridsdale HA, Hurst JR Dyspnoea perception and susceptibility to exacerbation in COPD. Thorax 2017;72:107–8. 10.1136/thoraxjnl-2016-209318 27815522

