# Correction: *Understanding changes in dyspnoea perception in obstructive lung disease after mindfulness training*

**DOI:** 10.1136/bmjresp-2018-000309corr1

**Published:** 2018-08-20

Malpass A, Feder G, Dodd JW. Understanding changes in dyspnoea perception in obstructive lung disease after mindfulness training. *BMJ Open Resp Res* 2018;5:e000309. doi: 10.1136/bmjresp-2018-000309

This article has been corrected since it first published. The open access license has been changed to:

This is an open access article distributed in accordance with the terms of the Creative Commons Attribution (CC BY 4.0) license, which permits others to distribute, remix, adapt and build upon this work, for commercial use, provided the original work is properly cited. See: http://creativecommons.org/licenses/by/4.0/

